# Investigation of Plasma-Electrolytic Processing on EDMed Austenitic Steels

**DOI:** 10.3390/ma16114127

**Published:** 2023-06-01

**Authors:** Timur Rizovich Ablyaz, Evgeny Sergeevich Shlykov, Karim Ravilevich Muratov, Ilya Vladimirovich Osinnikov, Mikhail Vladimirovich Bannikov, Sarabjeet Singh Sidhu

**Affiliations:** 1 Department of Mechanical Engineering, Perm National Research Polytechnic University, Perm 614000, Russia; kruspert@mail.ru (E.S.S.); karimur_80@mail.ru (K.R.M.); ilyuhaosinnikov@bk.ru (I.V.O.); 2 Institute of Continuous Media Mechanics of the Ural Branch, RAS, Perm 614013, Russia; mbannikov@icmm.ru; 3 Mechanical Engineering Department, Sardar Beant Singh State University, Gurdaspur 143521, Punjab, India

**Keywords:** electrical discharge machining, plasma electrolytic polishing, roughness, recast layer, residual stress, fatigue, chemical composition

## Abstract

This study investigates the effect of electrolytic plasma processing on the degree of defective layer removal from a damaged layer obtained after manufacturing operations. Electrical discharge machining (EDM) is widely accepted in modern industries for product development. However, these products may have undesirable surface defects that may require secondary operations. This work aims to study the die-sinking EDM of steel components followed by the application of plasma electrolytic polishing (PeP) to enhance the surface properties. The results showed that the decrease in the roughness of the EDMed part after PeP was 80.97%. The combined process of EDM and subsequent PeP makes it possible to obtain the desired surface finish and mechanical properties. In the case of finishing EDM processing and turning, followed by PeP processing, the fatigue life is enhanced without failure up to 10^9^ cycles. However, the application of this combined method (EDM + PeP) requires further research to ensure consistent removal of the unwanted defective layer.

## 1. Introduction

Conventional machining methods (mechanical processing methods), such as milling, stamping, and turning, are common technologies for manufacturing parts. However, significant wear of the cutting tool, high machining cost, and degradation of material surface properties occur when cutting hard materials. Furthermore, cutting along the trajectory of a complex profile requires additional advanced equipment, thus leading to an increase in the cost of producing parts. The effectiveness of conventional machining processes is constrained by the properties of the material and the complexity of the workpiece geometry [1,2,3,4,5].

Electrical discharge machining (EDM) provides an effective manufacturing technique to machine hard materials with an intrinsic geometry with improved physical and mechanical properties and dimensional accuracy. The low productivity of this process during the finishing phase hinders the development of this technology. It is difficult to obtain a high-finish substrate for EDM-treated surfaces [6,7].

Furthermore, the EDM process causes significant microstructural changes in the surface layer of the workpiece. The surface white (recast) layer may be defective/cracked due to uneven solidification under the shocks of the rapid heating and cooling stages of EDM. This results in declined mechanical properties of the base metal and cracks may occur under alternating loads. These cracks can propagate deep into a part leading to failure [8,9].

The EDM process parameters can significantly affect the thickness of the white (recast) layer. The magnitude of the current (I), the duration of the pulse (Pon), as well as voltage (V), determine the amount of energy supplied to the workpiece, causing the melting and evaporation of a larger volume of material. The spark energy (E) is the culmination of three parameters (such as V, I, Pon) and is calculated as follows [5]:

$$E\;=\;V\;\times \;I\;\times {\;P}_{\mathrm{on}}$$

where E = Energy (J); V = Voltage (V); I = Current (A); P_on_ = On-Time (µs) [10,11].

In this process, a tool electrode was used to machine the material by a series of electrical sparks generated in a dielectric medium and produce a replica of the tool profile. However, due to the rapid melting and cooling process and residual stresses, metallurgical transformations are generated on the machined surface after each discharge.

Residual stresses or lock-in stresses developed, owing to the rapid heating and cooling of the material of the part, which reduces the service life of the machined components [12]. These stresses are typically tensile in nature. If they exceed the yield strength of the material, it will cause surface cracking [12]. It is believed that the residual stresses were concentrated within the thickness of the white layer. These stresses are directly proportional to the values of the pulsed current and pulse duration (i.e., spark energy) [12,13,14].

Thus, the application of plasma electrolytic polishing (PeP) on complex-profile surfaces is the best solution to remove the defective recast layer after EDM. This technology is based on the electric discharge phenomena in the “metal-electrolyte” system and it is a special case of electrochemical machining that uses environmentally friendly electrolytes [15,16]. The workpiece is an anode connected to the DC supply [17,18,19,20].

Unlike other finishing methods, such as mechanical polishing, electrolytic-plasma polishing is based on plasma and electrochemical processes and the workpiece surface can be polished in a few minutes irrespective of its geometry. These processes occur due to the micro-explosions in a thin vapor-gas shell near the surface of a metal electrode immersed in a solution under high voltage [21]. The PeP process improves the surface properties and contours.

In the present study, the influence of PeP treatment on the degree of removal of a defective surface layer after EDM of austenitic steel is presented. The fatigue life, residual stresses, and surface finish of the component after PeP treatment were addressed.

## 2. Materials and Methods

**Figure 1:**
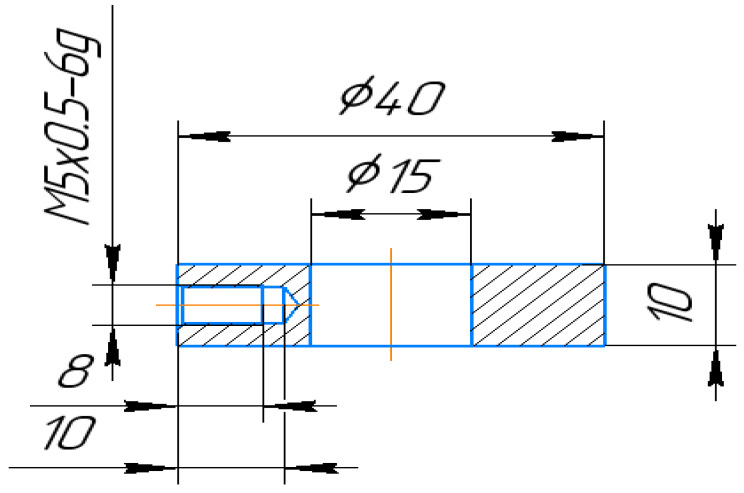
Copper ring electrode tool for EDM.

**Figure 2:**
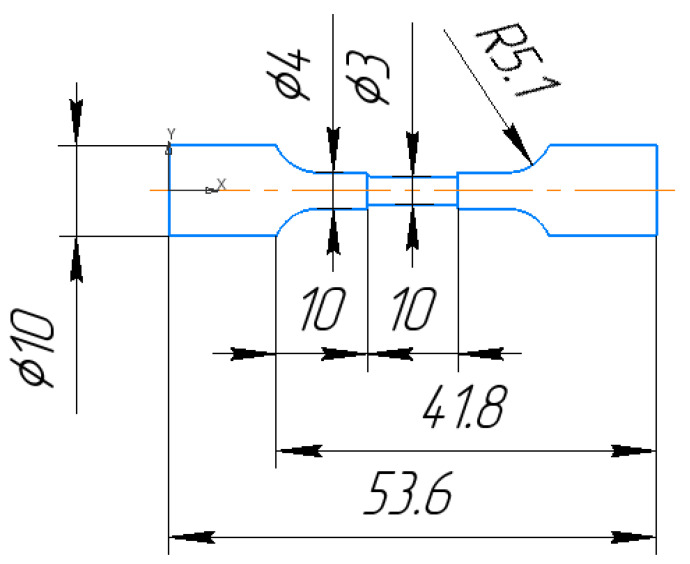
Schematic diagram of the experimental sample.

The main objective of the experimentation is to study the effect of using the combined technology of die-sinking EDM and subsequent plasma electrolytic polishing of austenitic steel (12Cr18Ni10Ti) on the properties of the surface (recast) layer of the workpiece (i.e., changes in the roughness of the treated surface; induced residual stresses; changes in fatigue life of samples). This selected steel is in demand in most industries as well as in the petrochemical industry. This steel composition is compatible with an aggressive environment. Ring electrode tools were made for the experimental runs (please refer to Figure 1). The material of the electrode tools is electrolytic copper. The workpiece (12Cr18Ni10Ti) was EDMed in the dog-bone-shaped geometry. The line diagram of the workpiece is shown in Figure 2.

The machining of the sample was performed using two different methods: electro-erosive (EDM) (non-conventional method) and turning (conventional method). The EDM was carried out on an Electronica Smart CNC copy-piercing EDM machine (Electronica Machine Tools, Pune, India) in the two modes of machining (i.e., roughing and finishing). The process parameters are listed in Table 1.

In this experimental design, a total of 18 samples were made, where six samples were processed using the EDM method in the rough mode and six samples were processed using the EDM method in the finishing mode. Six samples were processed using the turning operation. After processing via EDM and turning, half of the samples obtained by the methods were treated with the PeP method.

PeP was performed on a PeP-40 machine (Myprom, Minsk, Belarus) with a power of 40 kW. The method used was total immersion of the workpiece. The voltage at the electrodes was 380 V. The electrolyte used was a 5% ammonium sulfate solution ((NH_4_)_2_SO_4_). The solution was prepared by adding salt to the water and heated to 60 °C with constant stirring. The working temperature of the electrolyte was 85 °C. The processing time for the sample was 5 min.

The surface roughness of the processed surface was measured using a profilometer (Perthometer S2, Mahr GmbH, Goettingen, Germany). The base length was 0.8 mm. The following parameters were measured: average roughness height (Ra), maximum roughness height (Rmax), and average roughness step (Sm).

The turning of workpieces was carried out on a CNC turning-milling machine, Mazak HQR-100MSY (Mazak Company, Takeda, Oguchi-cho, Niwa-gun, Aichi-Pref., Japan). The Sandvik DDJNR 2020K 11 cutting tool, the DNMG 11 04 12-MM 2220 insert, was used. The turning parameters were a cutting speed of 120 mm/min, a feed rate of 0.15 mm/rev, a depth cut of 0.5 mm, and a spindle speed of 1200 rpm. The Amertend MSS Universal (Amertend, Ekaterinburg, Russia) was used as the coolant.

The residual stresses were measured in accordance with the methodology described in [22,23,24,25]. Residual stresses analysis was carried out by X-ray diffractometry using the Xstress 3000 robotic complex (Stresstech Oy, Jyväskylä, Finland) arrangements, considering the material parameters given in Table 2.

The parameters for measuring the residual stresses using Xstress 3000 are presented in Table 3.

Mathematical calculations for the residual stress measurements were carried out using the Xtronic diffractometer (Stresstech Oy, Jyväskylä, Finland) control program. The mathematical parameters are presented in Table 4.

**Figure 3:**
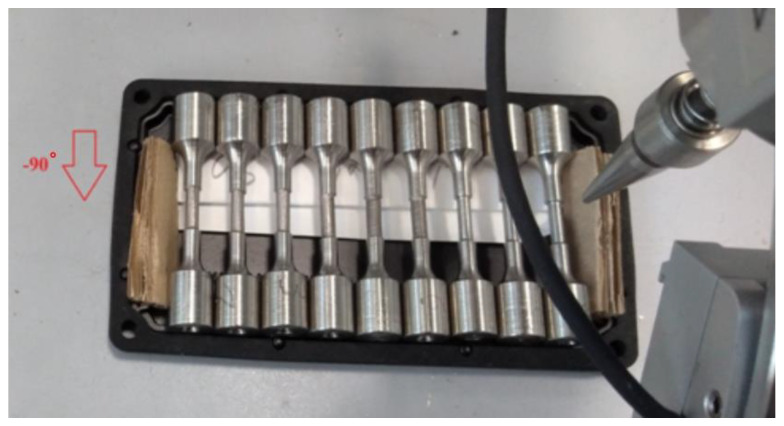
Residual stress measurement direction.

Stress measurements were performed on the surface of the machined part in the longitudinal direction (please refer to Figure 3).

**Figure 4:**
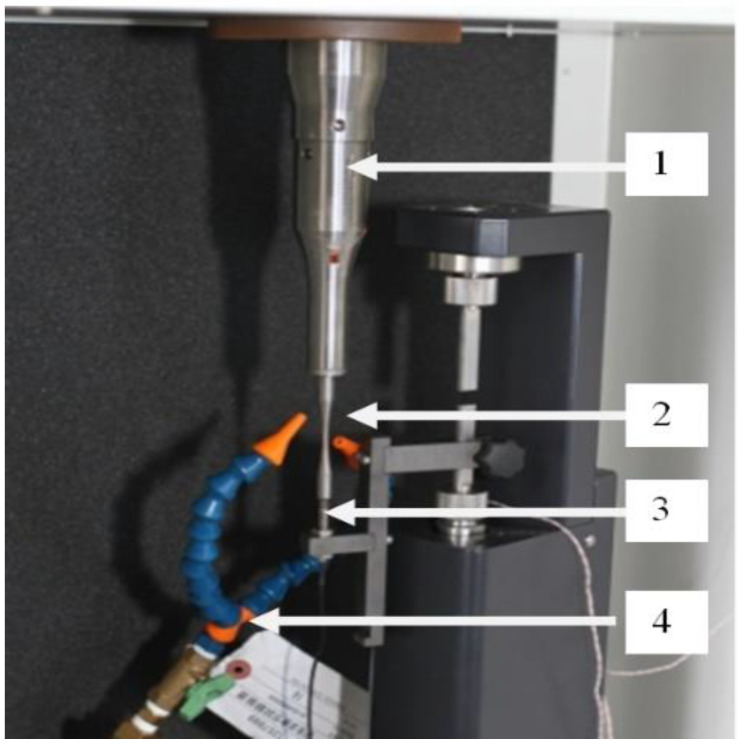
Pictorial view of the test system Shimadzu USF-2000: 1—Horn, 2—sample, 3—displacement sensor, 4—cooling system.

An ultrasonic machine Shimadzu USF-2000 (Shimadzu Europa GmbH, Kyoto, Japan) for testing the samples for fatigue life is shown in Figure 4. The principle of the ultrasonic analysis was that the generator creates electrical oscillations with a frequency of 20 kHz, which were converted into mechanical energy with the aid of piezoelectric crystal, which was further amplified by a waveguide and fed to the sample. The upper portion of the sample was attached to the waveguide, whereas the other end remained free. An air cooling system was used to prevent overheating.

**Figure 5:**
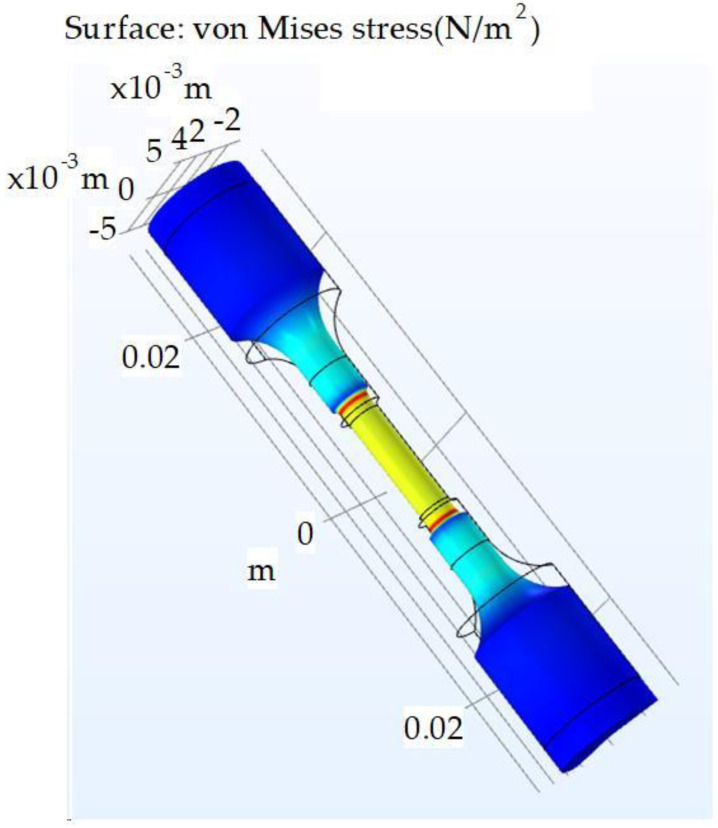
Stress distribution over the sample under tensile loading conditions.

**Figure 6:**
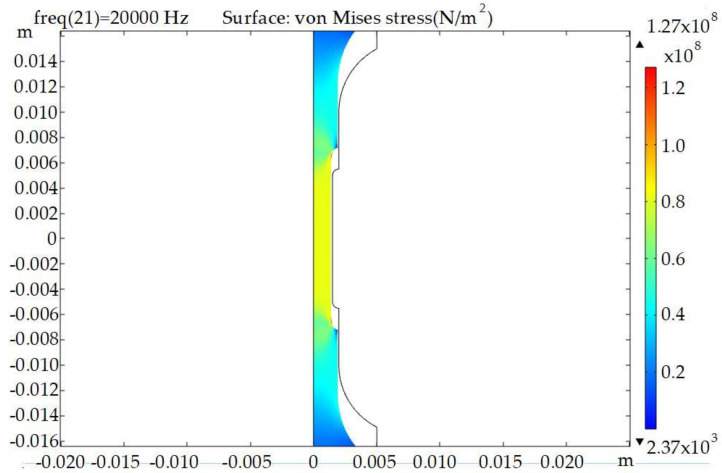
Stress with end displacements of the sample for 7.5 µm.

The testing machine was not capable of directly measuring the stress distribution while in the state of vibrations. The stress value was carried out by controlling the displacements (vibrations) of the end face of the sample using the analytical formulas described in [26]. Using the COMSOL Multiphysics 6.0 software mathematical package, the critical stress values in the samples under static loading were determined by considering their geometry (Figure 5 and Figure 6). Table 5 lists the stress values for the given displacement.

During the fatigue tests, it was observed that a fracture is carried out in places of stress concentration (i.e., at a minimum area of cross-section: red color zone in Figure 5).

**Figure 7:**
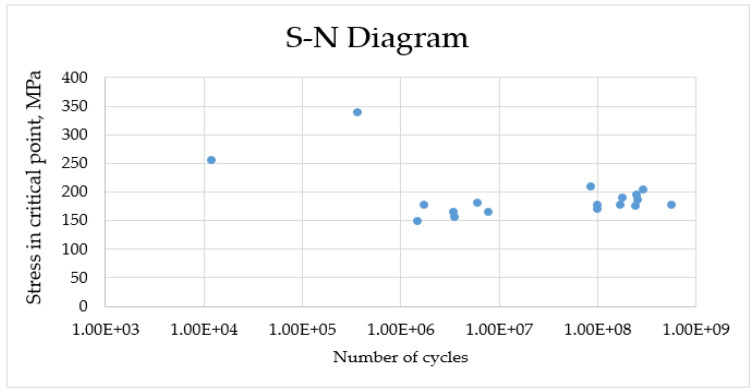
The dependence of stresses at a given displacement.

The stress distribution and displacement results are presented in Table 5. Based on the obtained data, the graph of the dependence of stresses on a given displacement was obtained (Figure 7).

**Figure 8:**
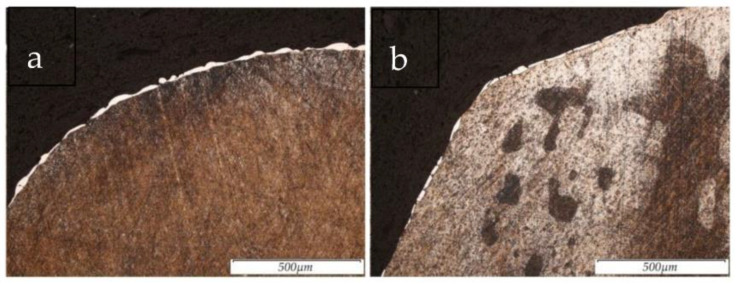
Sample structure at 100× magnification after: (**a**) EDM in rough mode. (**b**) EDM in rough mode and subsequent PeP.

The results of the cyclic tests are shown in the S-N diagram in Figure 8. The two clouds of points in Figure 7 are well separated at close values (150–200 MPa) in terms of the number of cycles to failure. In one case it was up to 10^7^ cycles and 10^8^. In both cases, a fatigue crack nucleates from the sample surface in the places with the maximum stress concentration, however, in samples with a rougher surface, the nucleation of the crack occurs two orders faster in magnitude. This is due to the fact that surface roughness, as well as uncompensated residual stresses caused by electrical discharge machining, create an additional stress concentration on the surface, thus, accelerating the fatigue crack formation. Plasma electrolytic polishing (PeP) reduces surface roughness and relieves some of the residual stresses, which leads to an increase in the fatigue properties of the material to its original value.

After testing for fatigue life, micro-sections were cut from the samples which were prepared in several stages: (i) the samples were first poured into Bakelite (BAKELITE SYNTHETICS Headquarters, Atlanta, GA, USA) and pressed in an automatic mounting press; (ii) grinding was performed on sandpaper with a grain size of p240 to p1500 on a Top Tech Plato grinding machine (Top tech machines co, Taichung City, Taiwan); (iii) after polishing, the sample was washed with water, degreased with a swab dipped in ethanol, and dried with filter paper; (iv) the micro-section was etched with Novikov’s solution to examine the microstructure of the sample.

The average depth of the white layer formed was measured. For this, several segments of length (L) were randomly placed on the cross-section of the layer, and the layer depth was calculated at a single point. The average thickness of the white layer was determined using the formula: Lav = (l1 + l2 + … + ln)/n, where l is the layer depth (mm) and n is the number of measurements. The measurement was carried out at least 30 times in the five most typical fields of view in each image.

The microstructure was studied using an OLYMPUS GX 51 light microscope at magnifications of 100×. The image measurements were processed using the OLYMPUS Stream 2.2 (Olympus corporation, Tokyo, Japan). Motion software. Further, scanning electron microscopy was used to determine the qualitative and quantitative compositions of the chemical elements in the surface layer. The measurements were performed using a REM-100U scanning electron microscope (Electron, Sumi, Russia).

## 3. Results

### 3.1. The Results of the Microstructure of Samples Processed via Various Processing Routes

The results of the surface structure of the steel 12Cr18Ni10Ti samples, obtained using the method of electrical discharge machining in the rough mode and after PeP, are shown in Figure 8a,b.

**Figure 9:**
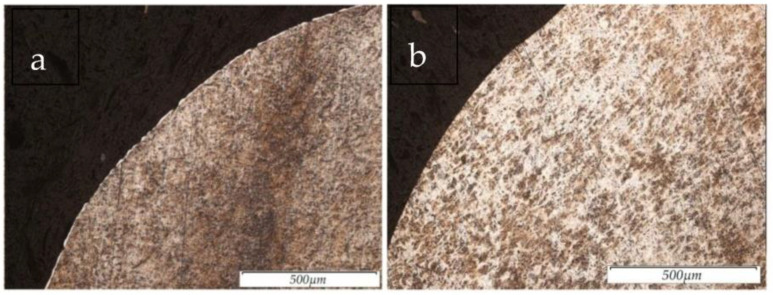
Sample structure at 100× magnification after: (**a**) EDM in the finishing mode and (**b**) EDM in the finishing mode and subsequent PeP.

The results of the surface structure of the steel 12Cr18Ni10Ti samples, obtained using electro-erosive machining in the finishing mode and subsequent PeP, are shown in Figure 9a,b.

**Figure 10:**
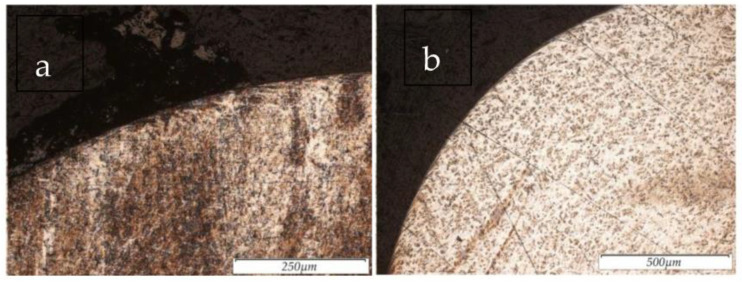
The structure of the samples at a magnification of 100× after: (**a**) turning and (**b**) turning and subsequent PeP.

The results of the surface structure of the steel 12Cr18Ni10Ti samples, obtained by turning, are shown in Figure 10a,b.

The average measured thickness of the white layer on the surface of the samples, obtained after EDM, is presented in Table 6.

As a result of the experiment, it was found that the removal of the defective (recast) layer from the surface of the EDMed samples occurs in the PeP process stage. It was also observed that surface defects were present on the samples machined via turning. This is due to the force exerted by the cutting tool on the sample. In the PeP process, the samples after EDM were in the finishing mode, and the complete removal of the white layer from the treated surface was observed. After rough EDM and PeP, the white layer from the sample was not completely removed. However, its thickness after the PeP process was reduced by the factor two times. The incomplete removal of the white layer may be due to the uneven removal of the recast layer during the PeP process [27].

### 3.2. Results of the Elemental Composition of the Surface Layer of the Samples

The results of the chemical composition of the samples are presented in Table 7. The elemental compositions of the samples were determined using energy-dispersive X-ray spectroscopy (EDAX)

The experimental results showed that EDM and PeP did not affect the elemental composition of steel 12Cr18Ni10Ti. No significant changes in the elemental composition were observed. The deposition of elements in the electrode tool material, working fluid, or solution was also not detected

### 3.3. The Results of Measuring the Roughness of the Machined Surface

**Figure 11:**
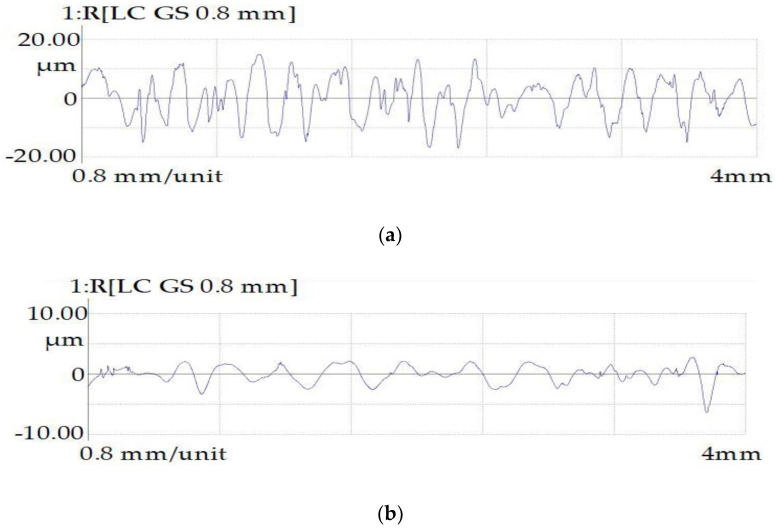
Surface profilograms of samples obtained by the rough EDM method: (**a**) before PeP and (**b**) after PeP.

The profiles of the surfaces of the samples obtained after rough EDM before and after PeP are shown in Figure 11.

**Figure 12:**
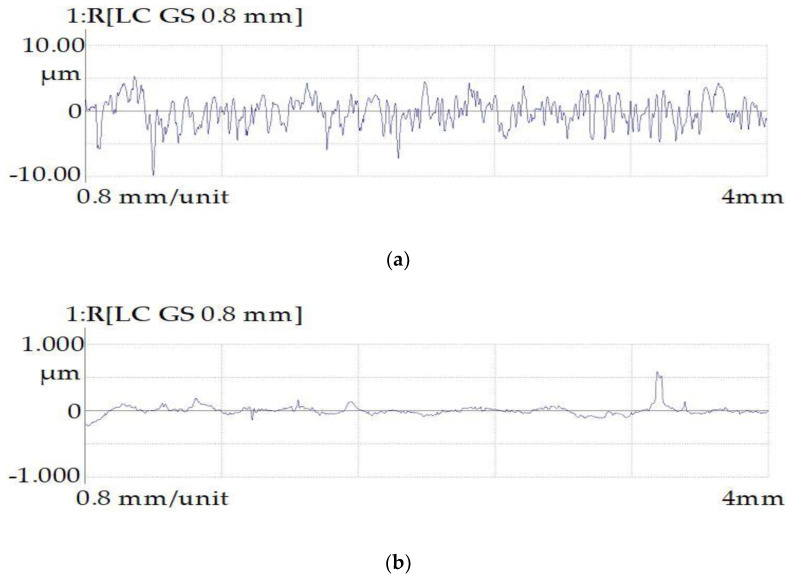
Surface profilograms of samples obtained by the finishing EDM method: (**a**) before PeP and (**b**) after PeP.

The surface profiles of the samples obtained by the EDM method before and after the EPT are shown in Figure 12.

**Figure 13:**
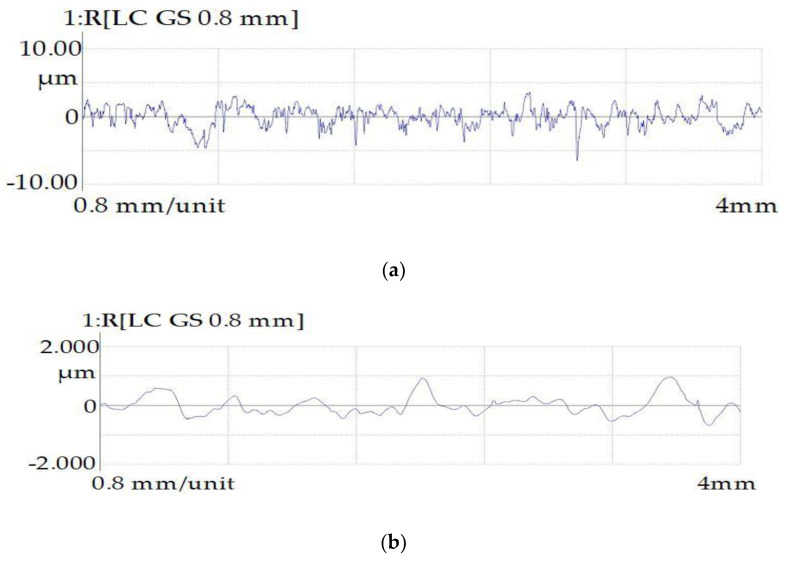
Surface profilograms of samples obtained by turning: (**a**) before PeP and (**b**) after PeP.

The surface profiles of the samples obtained after turning are shown in Figure 13.

The analysis of the surface topology indicated that the peaks and valleys obtained as a result of the EDM process, and the scratches that arose during the turning process, were smoothed after the application of the PeP process. Quantitative indicators of the surface roughness parameters of the samples are presented in Table 8.

**Figure 14:**
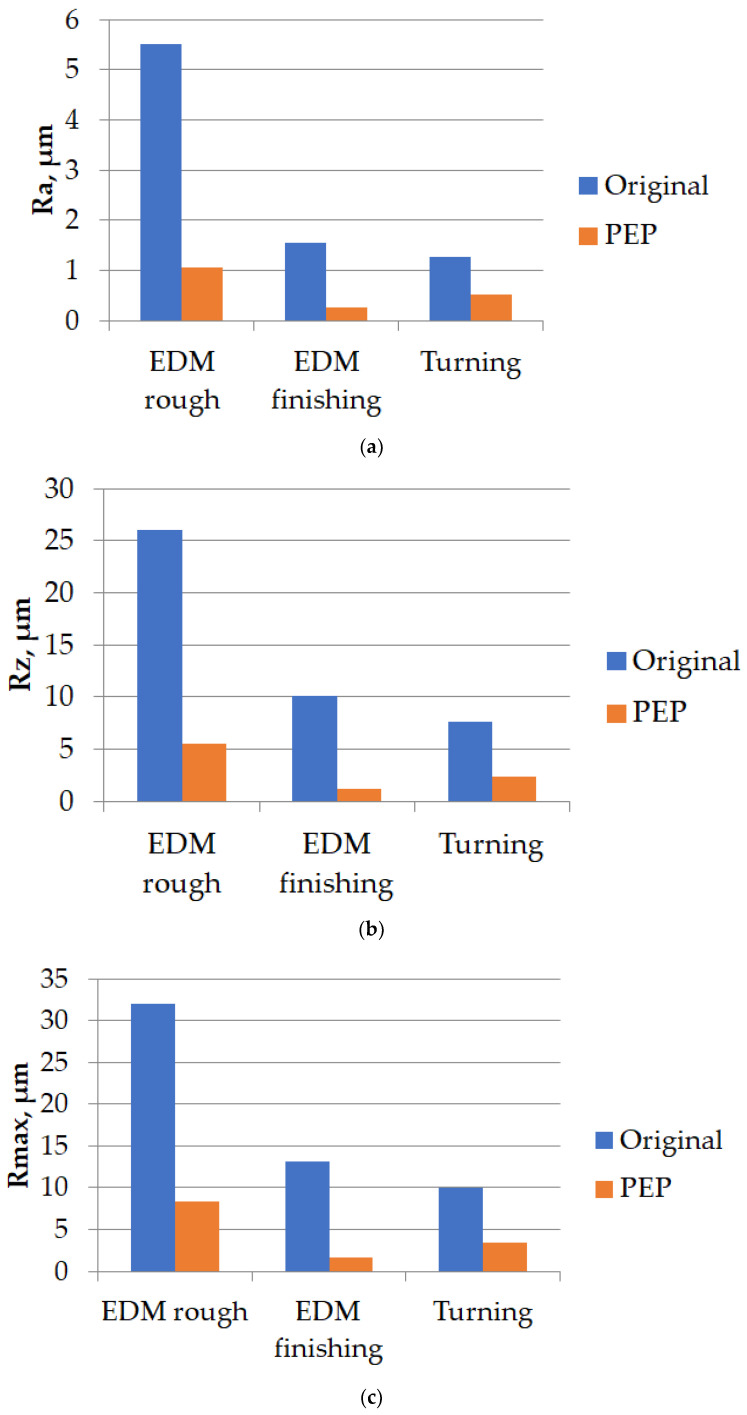

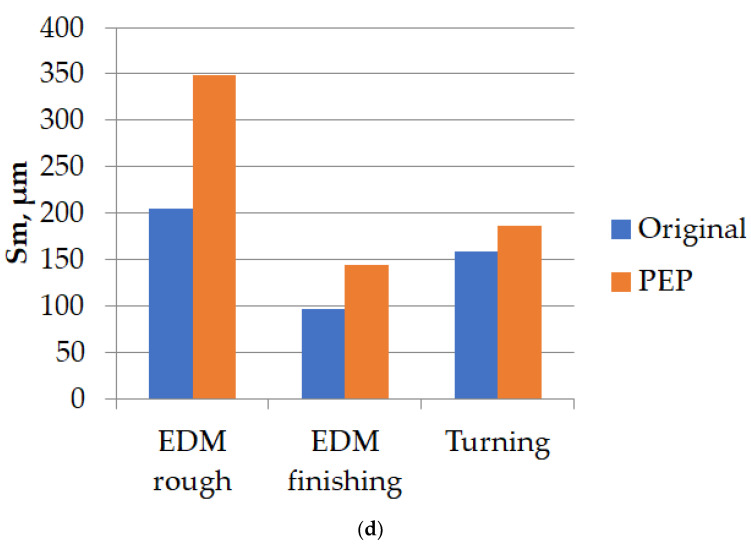
Surface roughness indicators before and after PeP: (**a**) Ra; (**b**) Rz; (**c**) Rmax; (**d**) Sm.

The graphs (Figure 14) were constructed to demonstrate the obtained data.

An analysis of the graphs (Figure 14) revealed that when processing the samples in the finishing mode, via turning and EDM methods, the roughness indices Ra, Rz, and Rmax are equivalent and the reading varies no more than 25%. However, the difference in the roughness step Sm was 40%. It has also been reported that productivity in the EDM finishing mode is lower. When EDM was used in the rough mode, the roughness indicators were much worse than those of the sample obtained using the turning method. The arithmetic mean deviation of the Ra profile is 4.3 times higher. The height of the profile for 10 points is 3.4 times higher. The maximum profile height is 3.2 times higher. In the process of PeP, a significant decrease in roughness (Ra, Rz, Rmax) occurs. The Ra parameter decreased by 5.2 times and the Rz parameter decreased by 4.7 times. The maximum profile height decreased by 3.8 times. The average roughness step Sm for 5 min of polishing was increased by 1.7 times. Thus, from working for 5 min, the PeP technology made it possible to obtain the roughness parameters better than the corresponding parameters of samples obtained using the turning method.

### 3.4. Results of Measurement of Residual Stresses on the Surface of Samples

The results of the residual-stress measurements on the surface of the samples are presented in Table 9.

**Figure 15:**
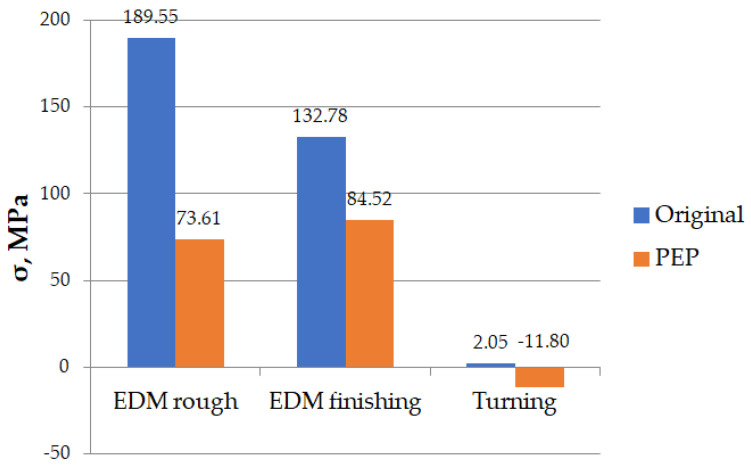
Comparison of residual stresses in various machining processes.

Based on the data obtained, a histogram of residual stresses was drawn for comparison (Figure 15)

An analysis of the graph (Figure 15) exhibits that tensile residual stresses arise during the EDM and PeP process. However, for the samples obtained using turning, small residual stresses, both tensile and compressive in nature, are observed. The application of the PeP process reduced the magnitude of residual stresses from the EDMed specimens. In the rough EDM mode, it decreases from 189.55 to 73.61 MPa (i.e., 61.6%), and in the finishing mode, it reduces from 132.78 to 84.52 MPa (i.e., 36.3%).

The ratio of the residual stresses before and after PeP for the samples after rough + PEP was significantly lower than that after finishing + PeP. This can be explained by the instability of the PeP process and the ease in the removal of uneven recast layers.

### 3.5. Fatigue Life Test Results

The results of testing samples for gigacycle fatigue life are presented in Table 10.

**Figure 16:**
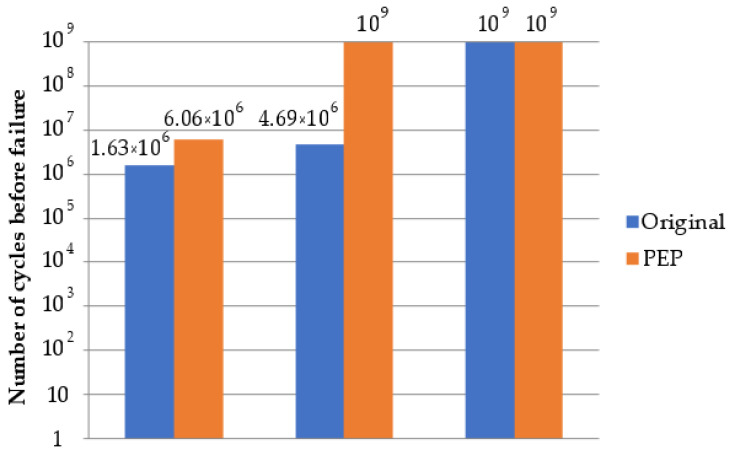
Gigacycle fatigue life test results.

A graph of the results of testing specimens for gigacycle fatigue life is shown in Figure 16.

An analysis of the data showed that the EDM process negatively influenced the resistance to cyclic fatigue failure. The samples obtained after EDM in the rough machining mode failed after an average of 1.63 million cycles. Samples obtained by the EDM in the finishing machining mode were a failure after an average of 4.69 million cycles. The large thickness of the defective (recast) layer is the cause of failure owing to the alleviation of the initiation of cracks or high-stress concentration in the modified phase alteration zone. The layer was not completely removed during the rough + PeP process. Nevertheless, the samples obtained using the turning method were not destroyed after 10^9^ cycles.

The PeP process increased the durability of the samples obtained using the EDM method in the rough machining mode up to 6.06 million cycles. The samples obtained by EDM in the finishing mode and turning were not destroyed or failed, owing to minimizing/eliminating the defective layer after the PeP process.

## 4. Conclusions

1. It has been established that a defective white (recast) layer is formed during the EDM process. In the productive rough machining mode, the thickness of the white layer is 3.3 times greater than in the finishing machining mode. PeP technology can significantly reduce the white (recast) thickness. It is advocated that for the complete removal of the white layer from the EDMed surface in the rough machining mode, the PeP processing time should be extended.
2. It is shown that the integrated EDM and subsequent PeP do not have a significant effect on the elemental composition of steel 12Cr18Ni10Ti. The elements deposition of the working fluid, electrolyte solution, and material from the electrode tool is not observed.
3. It has been established that during the EDM in the rough machining mode, the roughness indicators are significantly worse than those of the sample obtained using the turning method. The arithmetic mean deviation of the Ra profile is 4.3 times higher on the EDMed surface. In the process of the PeP process, there is a significant decrease in all roughness parameters (Ra, Rz, Rmax). The Ra parameter decreased by 5.2 times. The Rz parameter decreased by 4.7 times. The maximum profile height Rmax decreased by 3.8 times.
4. It is revealed that the maximum tensile residual stresses were obtained in the rough machining mode of the EDM. PeP technology reduces residual stresses by more than 2.5 times.
5. It is demonstrated that the EDMed samples in the rough machining mode failed after 1.63 × 10^6^ cycles when tested for gigacycle fatigue life. The samples obtained using the EDM method in the finishing mode failed after 4.69 × 10^6^ cycles. The samples obtained using the turning method are not destroyed. The failure in the EDMed sample is due to the presence of a white (recast) layer on the samples. The treatment of these samples with the PeP process considerably increased their fatigue life. Most of the samples obtained by the methods of EDM in the finish machining mode and turning are not destroyed/failed.
6. It is shown that EDM in rough mode and subsequent PeP makes it possible to obtain roughness and the mechanical properties proportional to the products obtained using the turning method. However, this combined technology requires additional research to stabilize the PeP process and to ensure consistent removal of the unwanted defective layers after the PeP process.

## Figures and Tables

**Table 1:** Processing modes of EDM.

Processing Mode	Current, I, A	Pulse Duration, Ton, µs	Voltage, U, V
Finish	2	40	50
Rough	8	150	100

**Table 2:** Material parameters.

Parameter	Value
Young’s modulus	198,000 MPa
Poisson’s ratio	0.30

**Table 3:** Residual stress measurement modes.

Parameter	Meaning
Method of measurement	modified “χ-method”
Collimator	2 mm (Dia)
Directions ϕ to the analysis point	−90°
Anode Tube	Cr
Vanadium filters	Not
Diffraction line (hkl)	(220)
Diffraction angle 2θ	148.9 deg
X-ray penetration depth at χ = 0°	6.3 µm
Exposure time in one goniometer position	65 s
Tilt angles χ	in the range [−30°;30°], are symmetrical in absolute values in both directions, where positive tilt angles are χ in the range [0°;30°] and negative tilt angles are χ in the range [−30°;0°]
Number of tilt angles ±χ	13, where N + χ = N − χ = 7 (including χ = 0° and assuming that the measurement at position χ = 0° is carried out once)
Oscillations of the X-ray beam (oscillation)	3 deg

**Table 4:** Parameters of mathematical processing of the results of measuring residual stresses.

Parameter	Meaning
Peak calculation	PeakFit Method
Peak level used for calculation	75
Subtraction of background radiation values	Linear
Setting 2θ angles	calibrated
Selecting anti-aliasing options	Not
Calculation of shear stresses	Elliptical Method
Calculation of principal stresses	Tridirectional method 0°, −45°, −90°
Stress tensor	Tridirectional method 0°, −45°, −90°

**Table 5:** Results of stress at critical zone w.r.t. displacement.

Displacement, µm	Stresses, MPa
5	53
10	107
15	160
20	214
30	321

**Table 6:** Thickness of the white layer on the surface of samples obtained using EDM.

Sample Processing Route	Thickness of the White Layer, µm
EDM (rough) + PeP	16.48
EDM (finishing) + PeP	-
EDM (rough)	32.09
EDM (finish)	9.42

**Table 7:** Elemental composition of the surface layer of the samples.

Type of Processing	Content of Elements, %					
	Fe	Cr	Ni	Mn	Si	Ti
Initial (turning)	base	17–19	9–11	≤2	≤0.8	0.4–1
PeP	base	17.2	9.9	1.3	0.6	0.6
EDM (finish)	base	18.1	9.9	1.1	0.7	0.4
EDM(rough)	base	17.6	10.1	1.2	0.7	0.6
EDM (finish) + PeP	base	18.8	9.5	1.2	0.7	0.5
EDM (rough) + PeP	base	18.4	9.6	1.0	0.8	0.5

**Table 8:** Sample roughness parameters.

Type of Sample Processing	Roughness Parameters, µm			
	Ra	Rz	Rmax	Sm
EDM (rough)	5.50	26.08	31.94	204.85
EDM (finish)	1.56	10.10	13.14	96.21
Turning	1.27	7.68	9.93	157.93
EDM (rough) + PeP	1.05	5.52	8.41	347.95
EDM (finish) + PeP	0.25	1.21	1.72	144.03
Turning + PeP	0.53	2.32	3.45	185.86

**Table 9:** Results of measurements of residual stresses.

Type of Processing	No. Sample	Values of Residual Stresses, MPa		
		Point 1	Point 2	Average
EDM (rough)	1	197.8	170.1	189.55
	2	180.7	158.4	
	3	213.3	217	
EDM (finishing)	4	137.8	131.3	132.78
	5	139.9	135.6	
	6	124.4	127.7	
Turning	7	14.3	−15	2.05
	8	24.1	−25.6	
	9	35.5	−21	
EDM (rough) + PeP	10	67.7	80.4	73.61
	11	70.35	75.4	
	12	68	79.8	
EDM (finishing) + PeP	13	105.6	110.6	84.52
	14	123.8	125.9	
	15	25.4	15.8	
Turning + PeP	16	−27.1	−33.4	−11.8
	17	−23.4	15.3	
	18	31.4	−33.6	

**Table 10:** Gigacycle fatigue life test results.

Type of Processing	No. Sample	Fracture Location	Number of Cycles to Failure	
EDM (rough)	1	Fillet	1.58 × 10^6^	1.63 × 10^6^
	2	Fillet	1.80 × 10^6^	
	3	Fillet	1.50 × 10^6^	
EDM (finishing)	4	Fillet	5.70 × 10^6^	4.69 × 10^6^
	5	Fillet	3.57 × 10^6^	
	6	Fillet	4.80 × 10^6^	
Turning	7	Not destroyed	10^9^	10^9^
	8	Not destroyed	10^9^	
	9	Not destroyed	10^9^	
EDM (rough) + PeP	10	Fillet	5.7 × 10^6^	6.06 × 10^6^
	11	Fillet	6.35 × 10^6^	
	12	Fillet	6.12 × 10^6^	
EDM (finishing) + PeP	13	Not destroyed	10^9^	10^9^
	14	Not destroyed	10^9^	
	15	Not destroyed	10^9^	
Turning + PeP	16	Not destroyed	10^9^	10^9^
	17	Not destroyed	10^9^	
	18	Not destroyed	10^9^	

## Data Availability

Not applicable.

## References

[1] Uthayakumar M., Prabhaharan G., Aravindan S., Sivaprasad J.V. (2008). Machining studies on bimetallic pistons with CBN tool using the Taguchi method—Technical communication. Mach. Sci. Technol..

[2] Uthayakumar M., Prabhakaran G., Aravindan S., Sivaprasad J.V. (2012). Influence of Cutting Force on Bimetallic Piston Machining by a Cubic Boron Nitride (CBN) Tool. Mater. Manuf. Process..

[3] Manikandan G., Uthayakumar M., Aravindan S. (2013). Machining and simulation studies of bimetallic pistons. Int. J. Adv. Manuf. Technol..

[4] Malakizadi I., Sadik L. (2013). Nyborg, Wear Mechanism of CBN Inserts During Machining of Bimetal Aluminum-grey Cast Iron Engine Block. Procedia CIRP.

[5] Mahajan A., Sidhu S.S. (2018). Enhancing biocompatibility of Co-Cr alloy implants via electrical discharge process. Mater. Technol..

[6] Han F., Jiang J., Yu D. (2007). Influence of discharge current on machined surfaces by thermo-analysis in finish cut of WEDM. Int. J. Mach. Tools Manuf..

[7] Pour G.T., Pour Y.T., Ghoreishi M. (2014). Thermal model of the electro-spark nanomachining process. Int. J. Mater. Mech. Manuf..

[8] Świercz R., Holubek R. (2020). Experimental investigation of influence electrical discharge energy on the surface layer properties after EDM. Weld. Technol. Rev..

[9] Liu J., Guo Y., Butler T., Weaver M. (2016). Crystallography, compositions, and properties of white layer by wire electrical discharge machining of nitinol shape memory alloy. Mater. Des..

[10] Klocke F., Hensgen L., Klink A., Ehle L., Schwedt A. (2016). Structure and composition of the white layer in the wire-EDM process. Procedia CIRP.

[11] Jose J., Shunmugam M. (2009). Investigation into white layer formed on wire electrical discharge machined Ti6Al4V surface. Int. J. Mach. Mach. Mater..

[12] Zeilmann R.P., Vacaro T., Zanotto F. (2013). Metallurgical alterations in the surface of steel cavities machined by EDM. Matéria.

[13] Shabgard M., Seyedzavvar M., Oliaei S. (2011). Influence of Input Parameters on the Characteristics of the EDM Process. Stroj.—J. Mech. Eng..

[14] Lee H., Rehbach W., Hsu F. (2004). The study of EDM hole-drilling method for measuring residual stress in SKD11 tool steel. J. Mater. Process. Technol..

[15] Nestler K., Böttger-Hiller F., Adamitzki W., Glowa G., Zeidler H., Schubert A. (2016). Plasma electrolytic polishing–An overview of applied technologies and current challenges to extend the polishable material range. Procedia CIRP.

[16] Danilov I., Hackert-Oschätzchen M., Zinecker M., Meichsner G., Edelmann J., Schubert A. (2019). Process understanding of plasma electrolytic polishing through mul-tiphysics simulation and inline metrology. Micromachines.

[17] Apelfeld A., Borisov A., Dyakov I., Grigoriev S., Krit B., Kusmanov S., Silkin S., Suminov I., Tambovskiy I. (2021). Enhancement of Medium-Carbon Steel Corrosion and Wear Resistance by Plasma Electrolytic Nitriding and Polishing. Metals.

[18] Zatkalíková V., Podhorský Š., Štrbák M., Liptáková T., Markovičová L., Kuchariková L. (2022). Plasma Electrolytic Polishing—An Ecological Way for Increased Corrosion Resistance in Austenitic Stainless Steels. Materials.

[19] Tavakoli H., Mousavi Khoie S.M., Rasooli F., Marashi S.P.H., Momeni F. (2015). Electrochemical and physical characteristics of the steel treated by plasma-electrolysis boronizing. Surf. Coat. Technol..

[20] Kumruoglu L.C., Ozel A. (2013). Plasma electrolytic saturation of 316L stainless steel in an aqueous electrolyte containing urea and ammonium nitrate. Mater. Technol..

[21] Wang J., Suo L.C., Guan L.L., Fu Y.L. (2012). Analytical study on mechanism of electrolysis and plasma polishing. Advanced Materials Research.

[22] Butt Z., Mehmood S., Sultan A., Anjum N., Anwar W. (2017). Determination of residual stress distribution in high strength aluminum alloy after EDM. Adv. Sci. Technol. Res. J..

[23] Aleksandrova O., Shiryaev A., Snegireva A., Trofimov V., Karmanov V. (2018). Influence of mechanical processing of steel 38KHN3MFA on the value of residual voltages. Vestn. PNRPU.

[24] Ablyaz T.R., Shlykov E.S., Muratov K.R., Osinnikov I.V. (2022). Study of the Structure and Mechanical Properties after Electrical Discharge Machining with Composite Electrode Tools. Materials.

[25] Sidhu S.S., Batish A., Kumar S. (2013). Neural network–based modeling to predict residual stresses during electric discharge machining of Al/SiC metal matrix composites. Proc. Inst. Mech. Eng. Part B J. Eng. Manuf..

[26] Bathias C., Paris P. (2010). Gigacycle fatigue of metallic aircraft components. Int. J. Fatigue.

[27] Vana D., Podhorsky S., Hurajt M., Hanzen V. (2013). Surface Properties of the Stainless Steel X10 CrNi 18/10 after Application of Plasma Polishing in Electrolyte. Int. J. Mod. Eng. Res..

